# A Cross-Sectional Study on Pediatric Resident Knowledge and Perceptions of Irritable Bowel Syndrome and Inflammatory Bowel Disease in Western Saudi Arabia

**DOI:** 10.7759/cureus.96583

**Published:** 2025-11-11

**Authors:** Ghassan A Sukkar, Reem E Kordi, Rafif Y Alahmadi

**Affiliations:** 1 Pediatric Gastroenterology, King Saud Bin Abdulaziz University for Health Sciences, Jeddah, SAU; 2 Pediatrics, Prince Mohammad bin Abdulaziz Hospital, Madinah, SAU; 3 Pediatrics, King Salman bin Abdulaziz Medical City, Madinah, SAU

**Keywords:** corticosteroids, crohn’s disease (cd), inflammatory bowel disease (ibd), irritable bowel syndrome (ibs), ulcerative colitis (uc)

## Abstract

Background: Irritable bowel syndrome (IBS) and inflammatory bowel disease (IBD) are chronic gastrointestinal disorders with overlapping features but different management needs. Little is known about how pediatric residents perceive these conditions.

Methods: A cross-sectional survey was conducted between January and April 2025 among pediatric residents in Western Saudi Arabia. A structured online questionnaire assessed knowledge, diagnostic approaches, and perceptions using case scenarios. The data were analyzed using descriptive and comparative statistics.

Results: A total of 197 residents completed the survey, with the majority aged 20-29 years. Nearly all identified IBS as an abdominal pain disorder (95.9%) and a diagnosis of exclusion (94.4%), but many showed gaps in applying the full diagnostic process. IBD was more often viewed as a critical condition requiring emergency referral (p<0.001), while IBS was perceived as less serious and frequently associated with symptom exaggeration. Psychological referral was endorsed for both conditions. Burnout was reported by 86.3% of residents, and junior trainees demonstrated greater adherence to updated diagnostic criteria.

Conclusion: Residents showed basic awareness of IBS and IBD but underestimated the impact of IBS. Improved training and strategies are necessary to address burnout and strengthen future clinical care.

## Introduction

Irritable bowel syndrome (IBS) is a functional gastrointestinal disorder characterized by chronic abdominal pain or discomfort and changes in bowel habits, with no detectable organic cause [1-3]. IBS can be diagnosed based on specific symptom criteria and by excluding other medical conditions [4]. Although the underlying mechanisms of IBS are not fully understood, diagnosis is based primarily on symptom presentation [2].

Inflammatory bowel disease (IBD), in contrast, is a chronic condition marked by unpredictable flare-ups, even during times of pharmacological remission. The ongoing nature of IBD often leads to multiple exacerbations and complications, necessitating treatments such as aminosalicylates (5-ASA), corticosteroids, immunosuppressants, biologics, and occasionally surgery. These treatments have a significant impact on the quality of life for young patients and their families [5]. IBD refers to conditions such as Crohn’s disease (CD) and ulcerative colitis (UC), where maladaptive immune responses, triggered by both genetic and environmental factors, result in chronic inflammation of the digestive system [6]. Differentiating between symptoms caused by inflammation and those that are functional is a key challenge in managing IBD [7].

IBS affects approximately 10-20% of people at any given time and has substantial financial implications, both in terms of direct healthcare expenses and lost productivity from missed work [4]. A meta-analysis estimated the global prevalence of IBS at 11.2% [2]. Meanwhile, the incidence of pediatric IBD has risen significantly in recent decades, with about 10% of IBD cases diagnosed in childhood [8].

Only one study has examined the prevalence of functional gastrointestinal symptoms in children with IBD as follows: in a cohort of 307 children with Crohn’s disease in remission, functional gastrointestinal symptoms were found in 6% of participants. The study hypothesized that the prevalence of IBS-like symptoms in children might be higher than the reported 6% [7].

The development of IBS may be linked to various factors, such as altered gut motility, increased intestinal sensitivity, and psychosocial influences. Additionally, IBS can arise following an episode of acute gastroenteritis, potentially due to lingering low-grade inflammation or immune system activity [4]. Due to the unclear causes, IBS is often classified as a “functional” disorder. Psychological factors, including stress, anxiety, and low mood, are commonly emphasized in its management [9]. On the other hand, IBDs have multifactorial causes, resulting from interactions among genetic, environmental, microbiome, and immune system factors. In Crohn’s disease (CD), inflammation can affect the entire GI tract and perianal region, presenting in patches with alternating healthy and inflamed tissue and potentially leading to complications like strictures or fistulas. Ulcerative colitis (UC), however, is limited to the colon, where continuous inflammation starts in the rectum and moves upward [8].

While both IBS and IBD have chronic, relapsing courses that significantly impact a patient’s quality of life and social functioning, inflammation in IBS is markedly less severe than in IBD. However, reduced gut microbiome diversity has been observed in both disorders [10,11]. Patients with persistent IBS symptoms often experience reduced physical function, lower quality of life, and higher healthcare costs, with factors like psychological distress, symptom severity, and social disruptions contributing to the condition’s chronicity [12,13]. A significant relationship has been found between parental knowledge and a child’s understanding of IBD, highlighting the importance of educational programs for both patients and families [5].

Although most IBS research involves patients referred to specialists, the majority of cases are managed by primary care physicians. There is strong support for diagnosing IBS based on typical symptoms, which minimizes the need for extensive testing and helps reduce associated healthcare costs [12,13]. This makes it essential for primary care providers to be proficient in recognizing IBS symptoms and managing its treatment effectively. Patients with IBS are encouraged to actively participate in managing their symptoms in collaboration with healthcare providers. Providers, in turn, must deepen their understanding of IBS, recognize its impact on patients’ daily lives, and provide empathetic, informative, and supportive care [14].

Unfortunately, patients with IBS are sometimes perceived as demanding or having unrealistic treatment expectations, leading to feelings that their condition is “less real” or taken less seriously by physicians. Conversely, organic disorders like IBD generally receive more validation from healthcare providers, who may view these conditions with fewer assumptions [11].

## Materials and methods

Methodology

This cross-sectional descriptive study aimed to assess the knowledge and perceptions of pediatric residents in the western region of Saudi Arabia regarding irritable bowel syndrome (IBS) and inflammatory bowel disease (IBD). Conducted between January and April 2025, the research targets residents enrolled in the Saudi Pediatric Residency Program within the specified region. Exclusion criteria encompass medical students, general practitioners, and residents training outside the western region.

Participants will be recruited through convenience sampling, utilizing digital communication platforms, such as email and WhatsApp (Mountain View, CA: Meta Platforms, Inc.), to distribute a structured online questionnaire (https://forms.gle/18nRhHMGJAZJep8G8). The sample size was determined to be 350 participants, calculated using an online tool with a 95% confidence level, a 5% margin of error, and an assumed prevalence of 50%.

The primary data collection instrument is a self-administered online questionnaire for hypothetical cases developed specifically for this study. It evaluates participants' knowledge of IBS and IBD, diagnostic confidence, and perceptions regarding the management of these conditions. To ensure content validity, the questionnaire was reviewed by a consultant in pediatric gastroenterology and will be hosted on a secure platform, Google Forms (Mountain View, CA: Google LLC), to maintain data confidentiality and accessibility. Data were exported and analyzed using the Statistical Package for the Social Sciences (SPSS) software (Armonk, NY: IBM Corp.). Descriptive statistics, including measures of central tendency and dispersion, will summarize demographic characteristics and response patterns.

A pilot test was done, and the average time required to complete the questionnaire was approximately 5-10 min; however, pilot data were excluded from the final analysis. Ethical approval for the study was obtained from the Research and Ethical Committee at King Salman bin Abdulaziz Medical City in Madinah, Saudi Arabia.

Statistical analysis

Statistical analysis was done by SPSS version 28 (Armonk, NY: IBM Corp.). Numerical data were presented as the mean and standard deviation (SD), analyzed within the same group using a paired t-test and between each two groups using an independent t-test, while across more than two groups using one-way ANOVA. Categorical data were presented as frequencies and percentages and analyzed using the chi-square test or Fisher’s exact test, as appropriate. A two-tailed p<0.05 was considered statistically significant.

## Results

A total of 197 pediatric residents, 92 males and 105 females, responded to our questionnaire; the majority of them, 193 pediatric residents (98%), belonged to the 20-29 years age group. Regarding residency level, it was the first, second, third, and fourth for 63 pediatrics residents (32%), 59 pediatrics residents (29.9%), 43 pediatrics residents (21.8%), and 32 pediatrics residents (16.2%), respectively. Most residents (191, 97%) had <5 years in practice, with 98 pediatrics residents (49.7%) practicing at the Makkah Maternity Children's Hospital. Notably, the most commonly intended sub-specialty was general pediatrics (195, 99%) (Table 1).

**Table 1:** Demographic data of pediatric residents (n=197).

Items	Frequency	Percentage
Age (years)		
20-29	193	98
30-39	4	2
Gender		
Male	92	46.7
Female	105	53.3
Residency level		
R1	63	32
R2	59	29.9
R3	43	21.8
R4	32	16.2
Years in practice		
<5	191	97
6-10	6	3
Center of residency		
East Jeddah General Hospital	43	21.8
King Fahad Armed Forces Hospital	10	5.1
Maternity Children's Hospital, Makkah	98	49.7
Maternity Children's Hospital, Taif	16	8.1
Maternity Children's Hospital, Madinah	2	1
King Salman Medical City	3	1.5
National Guard Hospital	23	11.7
Prince Mohammed bin Abdul Aziz Hospital	1	0.5
Security Forces Hospital	1	0.5
Intended sub-specialty		
General pediatrics	195	99
Other	2	1

**Figure 1:**
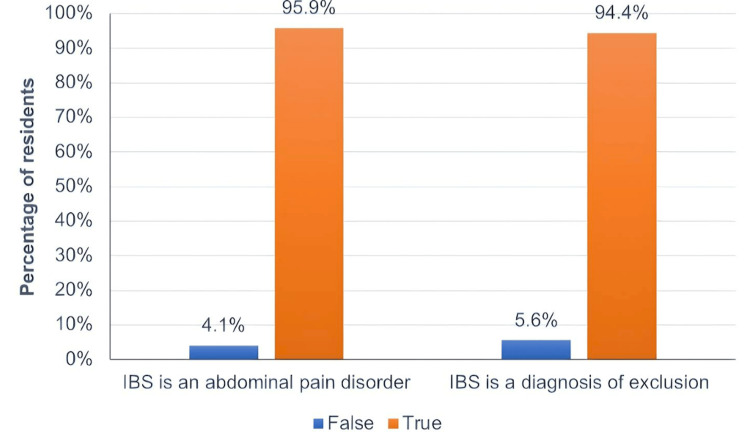
Pediatric residents’ knowledge of GI disorders. IBS: irritable bowel syndrome

As described in Table 2, the vast majority of residents correctly identified IBS as an abdominal pain disorder and a diagnosis of exclusion, 189 (95.9%) and 186 (94.4%), respectively. Regarding the IBS diagnosis method, it was correctly identified by 114 (57.9%) residents as involving history taking, physical examination, and clinical investigations. Further, 186 (94.4%) reported using diagnostic tools such as “Rome (no, yes, not sure) or Manning criteria to facilitate “IBS” diagnosis in their practice (Figure 1).

**Table 2:** Pediatric residents’ knowledge and attitudes towards GI disorders. IBS: irritable bowel syndrome

Items	Frequency	Percentage
IBS is an abdominal pain disorder		
False	8	4.1
True	189	95.9
IBS is a diagnosis of exclusion		
False	11	5.6
True	186	94.4
How would you diagnose IBS?		
By taking a history from the patient	9	4.6
By history taking and physical examination	73	37.1
By history taking, physical examination, and clinical investigations	114	57.9
Not sure	1	0.5
Have you ever used the diagnostic tools such as “Rome (no, yes, not sure) or Manning criteria” to facilitate “IBS” diagnosis in your practice?		
No	5	2.5
Yes	186	94.4
Not sure	6	3.0

For the IBS case presented in Table 3, 154 (78.2%) residents were likely to refer the patient to a psychologist/psychiatrist, and 161 (81.7%) believed that the patient was likely to be compliant with such a referral. Additionally, 168 (85.3%) were completely familiar with the condition, either personally or professionally, and, considering the criticality of the case, 70 (35.5%) answered correctly that it was stable. Moreover, 177 (89.8%) thought that the patient was likely to visit the ED, and 168 (85.3%) thought the patient was entirely responsible for the illness. Knowledge about the above-mentioned condition was deemed sufficient by 109 residents (55.3%) and by 58 residents (29.4%), representing 75% (148) and 100% (197) of the respective groups. It was considered a real illness by 174 (88.3%) residents. Among providers, 175 (88.8%) expressed confidence in their ability to treat the condition, while 170 (86.3%) believed that patients would adhere to the treatment plan successfully. Additionally, symptoms were likely to be exaggerated in the opinions of most residents (169, 85.8%), and expectations were likely to be reasonable in the views of 172 (87.3%) residents. Patience, optimism, resilience, and honesty were likely to be relevant to the condition, according to the opinions of 166 (84.3%), 159 (80.7%), 163 (82.7%), and 162 (82.2%) respondents, respectively.

**Table 3:** Pediatric residents’ attitude towards the following IBS case. IBS: irritable bowel syndrome

Items	Frequency	Percentage
IBS case: patient A presents with a one-year history of diffuse cramping abdominal pain that ranges in severity from mild to severe. While the pain started as infrequent and mild, more recently it has become almost daily and severe. They also report having persistent diarrhea since pain onset, producing multiple loose stools four times per day. Despite an extensive work-up in both outpatient and emergency room settings, including an endoscopic evaluation, a computed tomography (CT) of the abdomen and pelvis, routine blood work, and stool testing, no organic gastrointestinal disease was identified. As a result, they are diagnosed with irritable bowel syndrome diarrhea subtype (IBS-D).		
How likely would you refer this patient to a psychologist/psychiatrist?		
Disagree	11	5.6
Neutral	32	16.2
Agree	154	78.2
How likely do you feel this patient is to be compliant with the above referrals?		
Unlikely	4	2.0
Neutral	32	16.2
Likely	161	81.7
To what extent are you familiar with this condition, either personally or professionally?		
Not familiar	4	2.0
Neutral	25	12.7
Completely familiar	168	85.3
How critical do you think this patient is?		
Stable	70	35.5
Needs observation	118	59.9
Critical	6	3.0
Life threatening	3	1.5
Do you think this patient will ever visit the ED?		
Neutral	20	10.2
Likely	177	89.8
To what degree do you feel this patient is responsible for their illness?		
Not at all responsible	4	2.0
Neutral	25	12.7
Completely responsible	168	85.3
Do you feel like you have been taught enough about this condition in your medical education?		
25% have enough knowledge about the medical condition	8	4.1
50% have enough knowledge about the medical condition	22	11.2
75% have enough knowledge about the medical condition	109	55.3
100% have enough knowledge about the medical condition	58	29.4
Do you think that this is a “real” illness?		
Disagree	1	0.5
Neutral	22	11.2
Agree, it’s a real illness	174	88.3
As a provider, how confident are you that you would be able to treat this condition?		
Unlikely	1	0.5
Neutral	21	10.7
Confident	175	88.8
Do you think the patient will be able to successfully adhere to the treatment plan?		
Disagree	3	1.5
Neutral	24	12.2
Agree	170	86.3
To what extent do you feel this patient may be easy to get along with?		
Difficult	6	3.0
Neutral	26	13.2
Easy	165	83.8
How likely do you feel this patient will agree with your treatment plan?		
Unlikely	3	1.5
Neutral	30	15.2
Likely	164	83.2
To what extent do you feel this patient’s symptoms may be exaggerated?		
Unlikely	2	1.0
Neutral	26	13.2
Likely	169	85.8
How likely do you think this patient will have reasonable expectations?		
Unlikely	2	1.0
Neutral	23	11.7
Likely	172	87.3
How likely do you feel your patient is to demonstrate each of the following, relative to their condition?		
Patience		
Unlikely	4	2.0
Neutral	27	13.7
Likely	166	84.3
Optimism		
Unlikely	4	2.0
Neutral	34	17.3
Likely	159	80.7
Resilience		
Unlikely	4	2.0
Neutral	30	15.2
Likely	163	82.7
Honesty		
Unlikely	5	2.5
Neutral	30	15.2
Likely	162	82.2

For the IBD case, 162 (82.2%) residents were likely to refer that patient to a psychologist/psychiatrist, and 156 (79.2%) believed that the patient was likely to be compliant with that referral. Additionally, 155 (78.7%) were completely familiar with the condition, either personally or professionally, and, considering the criticality of the case, 108 (54.8%) correctly stated that it required observation. Moreover, 82.2% (162) thought that the patient was likely to visit the ED, and 84.8% thought the patient was completely responsible for the illness. Knowledge about the above-mentioned condition was considered sufficient by 105 residents (53.3%), representing 148 individuals (75%) in one group and 197 individuals (100%) in another, with 63 (32%) also deeming it adequate. It was considered a real illness by 180 (91.4%) residents. As providers, 176 (89.3%) residents were confident about their ability to treat that condition, 180 (91.4%) believed that the patient would successfully adhere to the treatment plan, 172 (87.3%) thought that the patient would be easy to get along with, and 173 (87.8%) thought they were likely to agree with the treatment plan. Symptoms were likely to be exaggerated in the opinions of most residents (173, 87.8%), and expectations were likely to be reasonable in the opinions of 176 (89.3%). Patience, optimism, resilience, and honesty were likely to be relevant to the condition, in the opinions of 174 (88.3%), 172 (87.3%), 170 (86.3%), and 172 (87.3%), respectively. Notably, 99 (50.3%) residents expected patients to be talking 100% of the time during the visit (Table 4).

**Table 4:** Pediatric residents’ attitudes towards the following IBD case. IBD: inflammatory bowel disease

Items	Frequency	Percentage
IBD case: patient B presents with a one-year history of episodes of worsening right lower quadrant abdominal pain that ranges from mild to severe. During these episodes, they also report having progressive diarrhea characterized by frequent loose stools, up to five per day. Through an extensive work-up, including colonoscopy, computed tomography (CT) of the abdomen and pelvis, routine blood work, and stool testing, they are diagnosed with ileo-colonic Crohn's disease.		
How likely would you to refer this patient to a psychologist/psychiatrist?		
Disagree	5	2.5
Neutral	30	15.2
Agree	162	82.2
How likely do you feel this patient is to be compliant with the above referrals?		
Unlikely	5	2.5
Neutral	36	18.3
Likely	156	79.2
To what extent are you familiar with this condition, either personally or professionally?		
Not familiar	4	2.0
Neutral	38	19.3
Completely familiar	155	78.7
How critical do you think this patient is?		
Stable	31	15.7
Needs observation	108	54.8
Critical	53	26.9
Life threatening	5	2.5
Do you think this patient will ever visit the ED?		
Unlikely	2	1.0
Neutral	33	16.8
Likely	162	82.2
To what degree do you feel this patient is responsible for their illness?		
Not at all responsible	13	6.6
Neutral	17	8.6
Completely responsible	167	84.8
Do you feel like you have been taught enough about this condition in your medical education?		
0% have enough knowledge about the medical condition	6	3.0
25% have enough knowledge about the medical condition	5	2.5
50% have enough knowledge about the medical condition	18	9.1
75% have enough knowledge about the medical condition	105	53.3
100% have enough knowledge about the medical condition	63	32.0
Do you think that this is a “real” illness?		
Disagree	2	1.0
Neutral	15	7.6
Agree, it’s a real illness	180	91.4
As a provider, how confident are you that you would be able to treat this condition?		
Unlikely	4	2.0
Neutral	17	8.6
Confident	176	89.3
Do you think the patient will be able to successfully adhere to the treatment plan?		
Disagree	1	0.5
Neutral	16	8.1
Agree	180	91.4
To what extent do you feel this patient may be easy to get along with?		
Difficult	5	2.5
Neutral	20	10.2
Easy	172	87.3
How likely do you feel this patient will agree with your treatment plan?		
Unlikely	3	1.5
Neutral	21	10.7
Likely	173	87.8
To what extent do you feel this patient’s symptoms may be exaggerated?		
Unlikely	6	3.0
Neutral	18	9.1
Likely	173	87.8
During a visit with the patient, what percent of the visit do you think they will be talking?		
25%	4	2.0
50%	10	5.1
75%	84	42.6
100%	99	50.3
How likely do you think this patient will have reasonable expectations?		
Unlikely	1	0.5
Neutral	20	10.2
Likely	176	89.3
How likely do you feel your patient is to demonstrate each of the following, relative to their condition?		
Patience		
Unlikely	2	1.0
Neutral	21	10.7
Likely	174	88.3
Optimism		
Unlikely	3	1.5
Neutral	22	11.2
Likely	172	87.3
Resilience		
Unlikely	4	2.0
Neutral	23	11.7
Likely	170	86.3
Honesty		
Unlikely	4	2.0
Neutral	21	10.7
Likely	172	87.3

**Figure 2:**
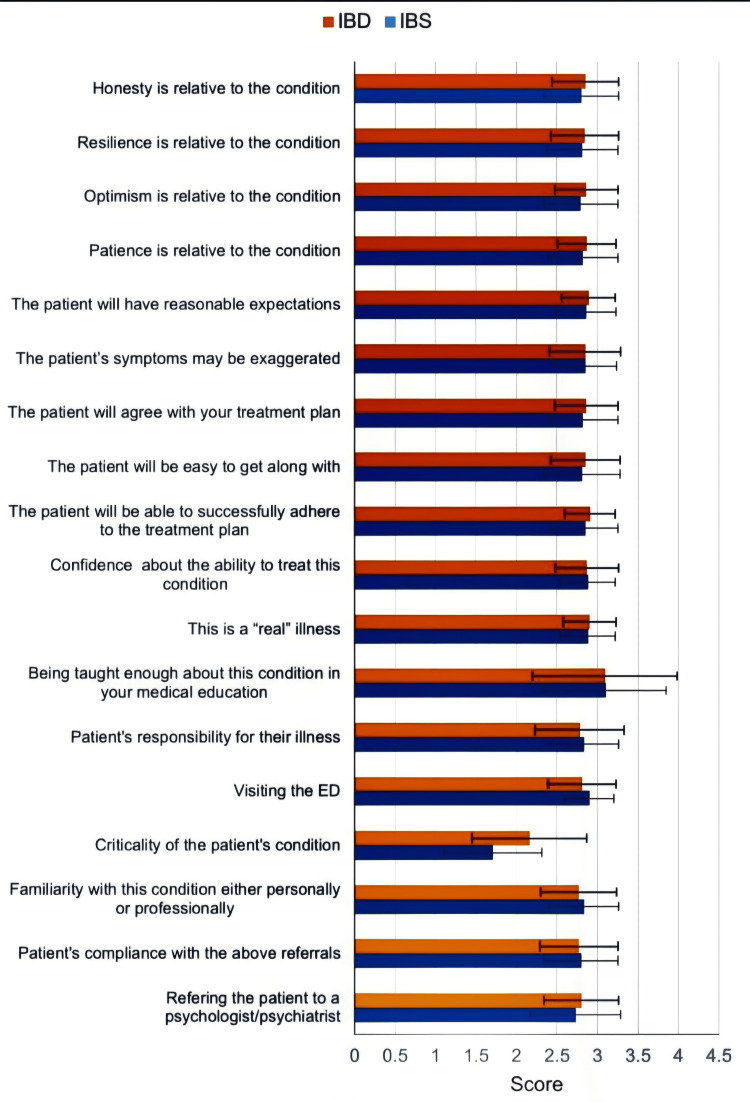
Residents’ perception of IBS and IBD. IBS: irritable bowel syndrome; IBD: inflammatory bowel disease

Overall, 170 residents (86.3%) reported being completely burned out from residency, and 173 (87.8%) felt that they had become more uncaring toward people since starting residency (Table 5). Residents perceived IBS as a less critical condition than IBD (p<0.001), with a greater tendency for IBS patients to visit the ED (p=0.017) (Table 6, Figure 2).

**Table 5:** Pediatric residents’ overall perception.

Items	Frequency	Percentage
I feel burned out from residency		
Not at all	4	2.0
Neutral	23	11.7
Completely	170	86.3
I have become more uncaring toward people since starting residency		
Not at all responsible	2	1.0
Neutral	22	11.2
Completely responsible	173	87.8

**Table 6:** Comparison between residents’ perception of IBS and IBD. Numerical data are presented as mean±SD and categorical data are presented as frequency (%). Statistical significance was set at p<0.05. IBS: irritable bowel syndrome; IBD: inflammatory bowel disease

Items	IBS	IBD	p-Value
How likely would you to refer this patient to a psychologist/psychiatrist?	2.73±0.56	2.8±0.46	0.122
How likely do you feel this patient is to be compliant with the above referrals?	2.8±0.45	2.77±0.48	0.487
To what extent are you familiar with this condition either personally or professionally?	2.83±0.43	2.77±0.47	0.091
How critical do you think this patient is?	1.71±0.6	2.16±0.71	<0.001
Do you think this patient will ever visit the ED?	2.9±0.3	2.81±0.42	0.017
To what degree do you feel this patient is responsible for their illness?	2.83±0.43	2.78±0.55	0.182
Do you feel like you have been taught enough about this condition in your medical education?	3.1±0.75	3.09±0.89	0.788
Do you think that this is a “real” illness?	2.88±0.34	2.9±0.33	0.425
As a provider, how confident are you that you would be able to treat this condition?	2.88±0.34	2.87±0.39	0.706
Do you think the patient will be able to successfully adhere to the treatment plan?	2.85±0.4	2.91±0.31	0.070
To what extent do you feel this patient may be easy to get along with?	2.81±0.47	2.85±0.43	0.195
How likely do you feel this patient will agree with your treatment plan?	2.82±0.43	2.86±0.39	0.190
To what extent do you feel this patient’s symptoms may be exaggerated?	2.85±0.39	2.85±0.44	>0.999
How likely do you think this patient will have reasonable expectations	2.86±0.37	2.89±0.33	0.436
How likely do you feel your patient is to demonstrate each of the following, relative to their condition?			
Patience	2.82±0.43	2.87±0.36	0.123
Optimism	2.79±0.46	2.86±0.39	0.052
Resilience	2.81±0.44	2.84±0.42	0.298
Honesty	2.8±0.46	2.85±0.41	0.109

There was a statistically significant relationship between residency level, residents’ experience, and knowledge of IBS (p=0.002 and 0.003, respectively). Most correct answers identifying IBS as a diagnosis of exclusion came from R1 and R2 residents. Specifically, 61 (32.8%) correct answers versus 2 (18.2%) incorrect answers were from first-year residents; 59 (31.7%) versus 0 (0%) from second-year residents; 40 (21.5%) versus three (27.3%) from third-year residents; and 26 (14%) versus six (54.5%) from fourth-year residents. In total, 182 (97.8%) correct answers and nine (81.8%) incorrect answers were obtained from residents with <5 years of practice, whereas four (2.2%) correct answers and two (18.2%) incorrect answers were obtained from those with 6-10 years of practice (Table 7).

**Table 7:** Relation between pediatric residents’ knowledge of IBS and demographic characteristics. Numerical data are presented as mean±SD and categorical data are presented as frequency (%). Statistical significance was set at p<0.05. IBS: irritable bowel syndrome

Items	IBS is an abdominal pain disorder		p-Value	IBS is a diagnosis of exclusion		p-Value
	False, n=8 (%)	True, n=189 (%)		False, n=11 (%)	True, n=186 (%)	
Age (years)						
20-29	8 (100)	185 (97.9)	>0.999	10 (90.9)	183 (98.4)	0.207
30-39	0 (0)	4 (2.1)		1 (9.1)	3 (1.6)	
Gender						
Male	1 (12.5)	91 (48.1)	0.069	7 (63.6)	85 (45.7)	0.247
Female	7 (87.5)	98 (51.9)		4 (36.4)	101 (54.3)	
Residency level						
R1	5 (62.5)	58 (30.7)	0.318	2 (18.2)	61 (32.8)	0.002
R2	1 (12.5)	58 (30.7)		0 (0)	59 (31.7)	
R3	1 (12.5)	42 (22.2)		3 (27.3)	40 (21.5)	
R4	1 (12.5)	31 (16.4)		6 (54.5)	26 (14)	
Years in practice						
<5	8 (100)	183 (96.8)	0.609	9 (81.8)	182 (97.8)	0.003
6-10	0 (0)	6 (3.2)		2 (18.2)	4 (2.2)	
Intended sub-specialty						
General pediatrics	8 (100)	187 (98.9)	>0.999	11 (100)	184 (98.9)	>0.999
Other	0 (0)	2 (1.1)		0 (0)	2 (1.1)	

The comparison between age groups regarding their perception of IBS showed that 20-29-year-old residents considered patients’ resilience and honesty to be more relevant to IBS than did the 30-39-year-old residents (p=0.011 and p=0.016, respectively). In comparison to the 30-39-year-old residents, the younger ones (20-29 years old) gave significantly more favorable perceptions towards IBD patients’ compliance with referrals (p=0.030), familiarity with the condition either personally or professionally (p=0.001), confidence in their abilities to treat the condition (p=0.001), and the likelihood of symptoms’ exaggeration (p<0.001) (Tables 8, 9).

**Table 8:** Relation between pediatric residents’ age and perception of IBS and IBD. Numerical data are presented as mean±SD, and categorical data are presented as frequency (%). Statistical significance was set at p<0.05. IBS: irritable bowel syndrome; IBD: inflammatory bowel disease

Items	IBS perception/age (years)		p-Value	IBD perception/age (years)		p-Value
	20-29	30-39		20-29	30-39	
How likely would you to refer this patient to a psychologist/psychiatrist?	2.73±0.55	2.5±1	0.415	2.8±0.45	2.5±1	0.588
How likely do you feel this patient is to be compliant with the above referrals?	2.8±0.44	2.5±1	0.588	2.78±0.48	2.25±0.5	0.030
To what extent are you familiar with this condition, either personally or professionally?	2.84±0.4	2.25±0.96	0.303	2.78±0.45	2±0.82	0.001
How critical do you think this patient is?	1.71±0.6	1.5±0.58	0.491	2.15±0.69	2.75±1.26	0.095
Do you think this patient will ever visit the ED?	2.9±0.3	2.75±0.5	0.323	2.81±0.42	2.75±0.5	0.764
To what degree do you feel this patient is responsible for their illness?	2.84±0.4	2.25±0.96	0.303	2.79±0.54	2.25±0.96	0.051
Do you feel like you have been taught enough about this condition in your medical education?	3.11±0.74	2.5±1	0.105	3.09±0.89	3±0.82	0.844
Do you think that this is a “real” illness?	2.88±0.34	2.75±0.5	0.452	2.9±0.33	3±0	0.555
As a provider, how confident are you that you would be able to treat this condition?	2.89±0.33	2.5±0.58	0.269	2.89±0.38	2.25±0.5	0.001
Do you think the patient will be able to successfully adhere to the treatment plan?	2.85±0.39	2.5±0.58	0.079	2.91±0.3	2.75±0.5	0.296
To what extent do you feel this patient may be easy to get along with?	2.82±0.45	2.25±0.96	0.321	2.85±0.42	2.5±0.58	0.099
How likely do you feel this patient will agree with your treatment plan?	2.83±0.4	2.25±0.96	0.313	2.87±0.39	2.75±0.5	0.556
To what extent do you feel this patient’s symptoms may be exaggerated?	2.85±0.39	2.75±0.5	0.612	2.87±0.41	2±0.82	<0.001
During a visit with the patient, what percent of the visit do you think they will be talking?	-	-	-	0.85±0.17	0.81±0.24	0.635
How likely do you think this patient will have reasonable expectations	2.88±0.35	2.25±0.96	0.282	2.89±0.33	2.75±0.5	0.401
How likely do you feel your patient is to demonstrate each of the following, relative to their condition?						
Patience	2.83±0.41	2.25±0.96	0.310	2.88±0.36	2.5±0.58	0.279
Optimism	2.8±0.44	2.25±0.96	0.336	2.87±0.39	2.5±0.58	0.065
Resilience	2.82±0.44	2.25±0.5	0.011	2.85±0.41	2.5±0.58	0.097
Honesty	2.81±0.46	2.25±0.5	0.016	2.86±0.4	2.5±0.58	0.081

**Table 9:** Relation between pediatric residents’ gender and perception of IBS and IBD. Numerical data are presented as mean±SD, and categorical data are presented as frequency (%). Statistical significance was set at p<0.05. IBS: irritable bowel syndrome; IBD: inflammatory bowel disease

IBS perception/gender		p-Value	IBD perception/gender		p-Value
Male	Female		Male	Female	
2.78±0.51	2.68±0.6	0.179	2.75±0.53	2.84±0.4	0.191
2.77±0.49	2.82±0.41	0.464	2.71±0.57	2.82±0.39	0.110
2.84±0.4	2.83±0.45	0.891	2.72±0.52	2.81±0.42	0.177
1.71±0.58	1.7±0.62	0.984	2.12±0.69	2.2±0.73	0.429
2.88±0.33	2.91±0.28	0.435	2.78±0.46	2.84±0.37	0.360
2.84±0.43	2.83±0.43	0.891	2.75±0.57	2.81±0.54	0.451
3.15±0.71	3.06±0.78	0.376	3.14±0.72	3.04±1.01	0.416
2.88±0.33	2.88±0.36	0.931	2.9±0.33	2.9±0.33	0.956
2.87±0.37	2.9±0.31	0.595	2.87±0.42	2.88±0.36	0.906
2.85±0.39	2.85±0.41	0.997	2.88±0.36	2.93±0.25	0.238
2.76±0.5	2.85±0.43	0.198	2.85±0.42	2.85±0.43	0.997
2.83±0.41	2.81±0.44	0.786	2.85±0.42	2.88±0.36	0.609
2.86±0.38	2.84±0.4	0.711	2.87±0.4	2.83±0.47	0.513
-	-	-	0.86±0.16	0.85±0.18	0.807
2.84±0.43	2.89±0.32	0.362	2.91±0.32	2.87±0.34	0.329
2.77±0.49	2.87±0.37	0.133	2.83±0.41	2.91±0.31	0.095
2.8±0.45	2.77±0.47	0.616	2.82±0.44	2.9±0.34	0.160
2.75±0.53	2.86±0.35	0.100	2.84±0.43	2.85±0.41	0.859
2.74±0.55	2.85±0.36	0.110	2.85±0.39	2.86±0.43	0.874

The relationship between residency level and perception of IBS and IBD is descriptively depicted in Tables 10, 11. For IBS, we noted a significant relation between residency level and perceptions of condition criticality (p=0.001), visiting the ED (p=0.017), responsibility of patients towards their illness (p=0.017), how much residents were taught about the condition in their medical education (p<0.001), how real the condition was thought to be (p=0.040), exaggeration of symptoms (p=0.029) and how likely optimism (p<0.001), resilience (p=0.013) and honesty (p=0.001) were thought to be relative to the condition. As for IBD, there was a significant relation between residency level and perceptions of referring the patient to a psychologist/psychiatrist (p=0.015), familiarity with the condition personally or professionally (p=0.012), criticality of condition (p=0.002), how much residents were taught in their medical education (p=0.001), how real the condition was believed to be (p=0.017), the percent of time at which patients would be talking during the visit (p=0.005), and how likely honesty was believed to be relative to the condition (p=0.010).

**Table 10:** Relation between residency level and perception of IBS. Numerical data are presented as mean±SD, and categorical data are presented as frequency (%). Statistical significance was set at p<0.05. IBS: irritable bowel syndrome

Items	IBS perception/residency level				p-Value
	R1	R2	R3	R4	
How likely would you to refer this patient to a psychologist/psychiatrist?	2.68±0.62	2.83±0.46	2.77±0.53	2.56±0.62	0.143
How likely do you feel this patient is to be compliant with the above referrals?	2.76±0.43	2.86±0.43	2.79±0.47	2.75±0.51	0.564
To what extent are you familiar with this condition, either personally or professionally?	2.84±0.37	2.9±0.36	2.74±0.49	2.81±0.54	0.341
How critical do you think this patient is?	1.63±0.49	1.92±0.6	1.74±0.73	1.41±0.5	0.001
Do you think this patient will ever visit the ED?	2.9±0.3	2.98±0.13	2.86±0.35	2.78±0.42	0.017
To what degree do you feel this patient is responsible for their illness?	2.83±0.42	2.97±0.18	2.72±0.5	2.75±0.57	0.017
Do you feel like you have been taught enough about this condition in your medical education?	2.76±0.76	3.12±0.65	3.4±0.69	3.34±0.75	<0.001
Do you think that this is a “real” illness?	2.83±0.38	2.97±0.18	2.91±0.29	2.78±0.49	0.040
As a provider, how confident are you that you would be able to treat this condition?	2.86±0.4	2.95±0.22	2.88±0.32	2.81±0.4	0.259
Do you think the patient will be able to successfully adhere to the treatment plan?	2.83±0.42	2.9±0.36	2.84±0.43	2.81±0.4	0.705
To what extent do you feel this patient may be easy to get along with?	2.78±0.46	2.88±0.42	2.79±0.51	2.75±0.51	0.520
How likely do you feel this patient will agree with your treatment plan?	2.76±0.47	2.9±0.36	2.84±0.37	2.75±0.51	0.251
To what extent do you feel this patient’s symptoms may be exaggerated?	2.76±0.47	2.97±0.18	2.81±0.45	2.84±0.37	0.029
During a visit with the patient, what percent of the visit do you think they will be talking?	-	-	-	-	-
How likely do you think this patient will have reasonable expectations	2.87±0.38	2.92±0.28	2.86±0.35	2.75±0.51	0.248
How likely do you feel your patient is to demonstrate each of the following, relative to their condition?					
Patience	2.86±0.4	2.92±0.28	2.74±0.54	2.69±0.54	0.053
Optimism	2.84±0.37	2.95±0.22	2.56±0.59	2.69±0.59	<0.001
Resilience	2.83±0.42	2.93±0.31	2.65±0.57	2.75±0.44	0.013
Honesty	2.78±0.49	2.95±0.22	2.58±0.63	2.84±0.37	0.001

**Table 11:** Relation between residency level and perception of IBD. Numerical data are presented as mean±SD, and categorical data are presented as frequency (%). Statistical significance was set at p<0.05. IBD: inflammatory bowel disease

IBD perception/residency level				p-Value
R1	R2	R3	R4	
2.81±0.43	2.92±0.28	2.77±0.53	2.59±0.61	0.015
2.71±0.55	2.9±0.3	2.65±0.57	2.78±0.42	0.052
2.76±0.47	2.9±0.3	2.74±0.54	2.56±0.56	0.012
2.1±0.69	2.41±0.62	2.16±0.61	1.84±0.88	0.002
2.81±0.47	2.9±0.3	2.72±0.45	2.78±0.42	0.191
2.81±0.5	2.88±0.46	2.74±0.58	2.59±0.71	0.111
2.73±0.99	3.19±0.75	3.28±0.88	3.34±0.7	0.001
2.87±0.42	3±0	2.91±0.29	2.78±0.42	0.017
2.86±0.43	2.95±0.22	2.86±0.41	2.78±0.49	0.245
2.87±0.38	2.98±0.13	2.88±0.32	2.88±0.34	0.171
2.84±0.45	2.9±0.36	2.81±0.5	2.81±0.4	0.723
2.78±0.52	2.97±0.18	2.86±0.35	2.84±0.37	0.061
2.81±0.5	2.93±0.31	2.79±0.47	2.84±0.45	0.333
0.79±0.2	0.88±0.14	0.89±0.15	0.88±0.17	0.005
2.9±0.35	2.93±0.25	2.81±0.39	2.88±0.34	0.335
2.87±0.38	2.92±0.34	2.88±0.32	2.78±0.42	0.413
2.87±0.38	2.93±0.25	2.81±0.5	2.75±0.44	0.158
2.76±0.56	2.93±0.25	2.81±0.39	2.88±0.34	0.140
2.78±0.55	3±0	2.79±0.41	2.81±0.4	0.010

Residents with less experience (<5 years of practice) elicited significantly more positive perception of IBS patients’ agreement with the treatment plan than those with 6-10 years of practice (p=0.004) but the latter thought that IBD patients were significantly more likely to visit the ED (p<0.001) and more likely to be talking during the visit (p=0.047) (Table 12). There was no statistically significant relation between overall burnout of residents and age, gender, residency level, years of experience, and intended sub-specialty (Table 13).

**Table 12:** Relation between pediatric residents’ years of practice and perception of IBS and IBD. Numerical data are presented as mean±SD, and categorical data are presented as frequency (%). Statistical significance was set at p<0.05. IBS: irritable bowel syndrome; IBD: inflammatory bowel disease

Items	IBS perception/years in practice		p-Value	IBD perception/years in practice		p-Value
	<5	6-10		<5	6-10	
How likely would you to refer this patient to a psychologist/psychiatrist?	2.73±0.56	2.67±0.52	0.793	2.8±0.46	2.67±0.52	0.485
How likely do you feel this patient is to be compliant with the above referrals?	2.8±0.45	2.83±0.41	0.842	2.77±0.48	2.67±0.52	0.607
To what extent are you familiar with this condition, either personally or professionally?	2.84±0.41	2.5±0.84	0.362	2.77±0.47	2.5±0.55	0.159
How critical do you think this patient is?	1.71±0.6	1.5±0.55	0.397	2.18±0.69	1.67±1.21	0.082
Do you think this patient will ever visit the ED?	2.91±0.29	2.67±0.52	0.309	2.81±0.42	3±0	<0.001
To what degree do you feel this patient is responsible for their illness?	2.83±0.43	2.83±0.41	0.996	2.78±0.56	2.83±0.41	0.817
Do you feel like you have been taught enough about this condition in your medical education?	3.09±0.75	3.33±0.52	0.443	3.07±0.89	3.5±0.55	0.246
Do you think that this is a “real” illness?	2.88±0.34	2.83±0.41	0.746	2.9±0.33	3±0	0.467
As a provider, how confident are you that you would be able to treat this condition?	2.89±0.33	2.67±0.52	0.339	2.87±0.39	2.83±0.41	0.801
Do you think the patient will be able to successfully adhere to the treatment plan?	2.85±0.4	2.67±0.52	0.262	2.91±0.3	2.83±0.41	0.542
To what extent do you feel this patient may be easy to get along with?	2.81±0.47	2.83±0.41	0.889	2.85±0.43	2.83±0.41	0.933
How likely do you feel this patient will agree with your treatment plan?	2.83±0.41	2.33±0.52	0.004	2.87±0.38	2.67±0.52	0.207
To what extent do you feel this patient’s symptoms may be exaggerated?	2.85±0.39	2.83±0.41	0.927	2.85±0.44	2.83±0.41	0.935
During a visit with the patient, what percent of the visit do you think they will be talking?	-	-	-	0.85±0.17	0.96±0.1	0.047
How likely do you think this patient will have reasonable expectations	2.87±0.37	2.67±0.52	0.191	2.89±0.33	2.83±0.41	0.681
How likely do you feel your patient is to demonstrate each of the following, relative to their condition?						
Patience	2.82±0.43	2.83±0.41	0.950	2.88±0.36	2.67±0.52	0.158
Optimism	2.79±0.46	2.83±0.41	0.801	2.86±0.39	2.83±0.41	0.877
Resilience	2.81±0.44	2.67±0.52	0.433	2.84±0.42	2.83±0.41	0.956
Honesty	2.8±0.46	2.83±0.41	0.845	2.86±0.41	2.67±0.52	0.258

**Table 13:** Relation between pediatric residents’ burnout and demographic characteristics. Numerical data are presented as mean±SD, and categorical data are presented as frequency (%). Statistical significance was set at p<0.05.

Items	Burnout score	p-Value
Age (years)		
20-29	2.84±0.42	0.195
30-39	2.75±0.5	
Gender		
Male	2.87±0.4	0.398
Female	2.82±0.43	
Residency level		
R1	2.86±0.47	0.268
R2	2.9±0.3	
R3	2.84±0.37	
R4	2.72±0.52	
Years in practice		
<5	2.85±0.4	0.349
6-10	2.5±0.84	
Intended sub-specialty		
General pediatrics	2.85±0.41	0.244
Other	2.5±0.71	

## Discussion

This cross-sectional study aimed to assess the knowledge level and perceptions of pediatric residents regarding irritable bowel syndrome (IBS) and inflammatory bowel disease (IBD) in Western Saudi Arabia. Our study provides valuable insights into how future pediatric specialists view and approach these two commonly encountered gastrointestinal conditions, highlighting several areas of adequate knowledge as well as clinical deficiencies and misperceptions that may impact patient practice and care.

Most residents in this study demonstrated reasonable awareness of the clinical definitions of IBS and IBD, with the majority correctly identifying IBS as a functional abdominal pain disorder and a diagnosis of exclusion. This aligns with contemporary definitions as per the Rome IV criteria, which emphasize symptom-based diagnosis in the absence of red flags and alarming signs. However, a significant portion (over 40%) unfortunately failed to fully acknowledge the essential components of the diagnostic approach, including detailed history taking, physical examination, and selective investigations. Similar deficiencies were reported in the study by Al-Hazmi, where over one-third of physicians were uncertain about how to diagnose IBS, and fewer than a quarter utilized the Rome or Manning criteria [1].

A promising finding in our study was that most residents agreed that IBS is a real illness, rejecting outdated psychosomatic views. However, perceptions of severity and legitimacy continued to differ between IBS and IBD. Participants were more likely to view IBD as a critical condition requiring emergency visits and specialist referral, whereas IBS was perceived as less urgent or in need of medical attention. These findings are consistent with international literature. Henick et al. found that medical students and junior doctors exhibited more empathy and urgency in managing IBD compared to IBS, often viewing the latter as a psychosomatic or exaggerated illness [11].

This discrepancy in perception is not trivial. It reflects a deeper bias in how functional disorders are internalized within medical training. Halpert emphasized that patients with IBS frequently experience stigma from healthcare providers, leading to mistrust and dissatisfaction [14]. These provider-based biases can undermine the therapeutic alliance and contribute to unnecessary testing or delayed diagnosis. Our finding that a significant portion of residents questioned the need for urgent management or emergency evaluation for IBS supports this trend.

The knowledge-performance gap was further evident in the testing of associations between training level and diagnostic insight. Interestingly, junior residents (R1-R2) and those with fewer than five years of experience demonstrated a statistically significantly higher tendency to recognize IBS as a diagnosis of exclusion. This may reflect the impact of updated medical school curricula or recent continuing medical education (CME) initiatives. In contrast, senior residents (R3-R4) were less likely to apply these principles, suggesting that knowledge may weaken over time or that organic pathologies may be prioritized during clinical rotations. A similar trend was identified by Longstreth and Burchette, who reported that recent graduates were more likely to adopt guideline-based IBS diagnosis than senior practitioners who depended on outdated heuristics [13].

The clinical manifestations of IBS and IBD overlap, further complicating the diagnostic process, especially in pediatric populations. Diederen et al. reported a high prevalence of IBS-like symptoms in children with IBD in remission, which supports the need for diagnostic precision and confidence [7]. Without clear and structured diagnostic training, residents may conduct unnecessary investigations, such as colonoscopies and radiologic imaging, and misallocate resources. This was evident in our study, where many participants were unclear on whether IBS warranted emergency referral.

Another important dimension of our findings relates to the psychosocial component of both disorders. Despite strong evidence linking mental health issues, such as anxiety and depression, with IBS and IBD, the referral of patients to mental health professionals remains underutilized. Only 78.2% of residents recommended psychological referral for IBS compared to 82.2% for IBD. While this gap is small, it highlights an enduring discomfort in managing psychological comorbidities among future clinicians. Huisman et al. demonstrated that patients with IBS often feel dismissed when the emotional or cognitive dimensions of their illness are minimized, suggesting that integrated care models should be emphasized in training [6].

Burnout among residents was another significant finding, with over 86% reporting symptoms of complete burnout. This aligns with prior literature suggesting that burnout impairs empathy, increases diagnostic errors, and reduces patient satisfaction. Panagioti et al. identified burnout as a strong predictor of lower professionalism, which can impact how chronic conditions, such as IBS, are perceived and managed [15]. Incorporating well-being and resilience training into residency curricula may therefore enhance both clinician performance and patient care.

When comparing our findings to Al-Hazmi’s study, several parallels emerge. Both studies identified a foundational understanding of IBS among physicians, accompanied by important misconceptions and variable diagnostic practices. In Al-Hazmi’s study, only 36.9% of physicians correctly recognized the non-alarming features of IBS, and 35.4% were unsure how to approach the diagnosis, which is very similar to the diagnostic hesitations observed among our residents. Moreover, in both cohorts, continuing medical education (CME) was cited as a preferred method for bridging knowledge gaps. This consensus highlights the persistent need for structured educational modules, simulation-based learning, and interdisciplinary collaboration [1].

This study found that residents were more confident managing IBD in a structured clinical pathway, while IBS remained ambiguously handled. This could lead to a delay in accurate diagnosis and reassurance of such conditions, which can, in turn, lead to unnecessary healthcare utilization, as evidenced by Spiegel et al. and others [16].

In light of these findings, we recommend several practical interventions as follows: pediatric residency programs in Saudi Arabia should reinforce structured IBS/IBD modules that include both clinical and psychosocial components. Active use of case-based discussions and assessment modules is encouraged. Institutional burnout mitigation strategies should be deployed to safeguard resident performance and empathy. Lastly, national initiatives should be established to develop and disseminate Saudi-specific clinical guidelines on IBS and IBD to unify training and practice standards across the Kingdom.

## Conclusions

This study reveals that pediatric residents in Western Saudi Arabia possess a reasonable understanding of IBS and IBD; however, significant gaps remain, particularly in the perception and management of IBS. While IBD is consistently recognized as a serious condition, IBS is often undervalued and seen as less critical, despite its significant impact on patients’ lives. These perceptions, combined with the high rates of burnout among residents, highlight the need for better training and support. Strengthening residency education on functional gastrointestinal disorders and addressing physician well-being are key steps toward improving diagnostic confidence and delivering more balanced, patient-centered care.
