# Correction: A Cross-Sectional Study on Pediatric Resident Knowledge and Perceptions of Irritable Bowel Syndrome and Inflammatory Bowel Disease in Western Saudi Arabia

**DOI:** 10.7759/cureus.c425

**Published:** 2026-04-17

**Authors:** Ghassan A Sukkar, Reem E Kordi, Rafif Y Alahmadi

**Affiliations:** 1 Pediatric Gastroenterology, King Saud Bin Abdulaziz University for Health Sciences, Jeddah, SAU; 2 Pediatrics, Prince Mohammad bin Abdulaziz Hospital, Madinah, SAU; 3 Pediatrics, King Salman bin Abdulaziz Medical City, Madinah, SAU

This article has been corrected to change the affiliation of Dr. Ghassan Sukkar from Pediatric Gastroenterology, Hepatology, Nutrition, King Abdulaziz Medical City, Jeddah, SAU to Pediatric Gastroenterology, King Saud Bin Abdulaziz University for Health Sciences, Jeddah, SAU.

